# A systematic review of the clinical characteristics and course of atrioventricular blocks in hyperthyroidism

**DOI:** 10.1080/07853890.2024.2365405

**Published:** 2024-06-21

**Authors:** Fateen Ata, Haseeb Ahmad Khan, Hassan Choudry, Adeel Ahmad Khan, Shuja Tahir, Tiago Lemos Cerqueira, Ben Illigens

**Affiliations:** a Department of Endocrinology, Hamad General Hospital, Hamad Medical Corporation, Doha, Qatar; b Department of Clinical Research, Dresden International University, Dresden, Germany; c Department of Internal Medicine, Nishtar Medical College and Hospital, Multan, Pakistan; d Department of Internal Medicine, University Hospital of Coventry and Warwickshire, Coventry, UK; e Department of Cardiothoracic Surgery, St James’s Hospital, Dublin, Ireland

**Keywords:** Hyperthyroidism, Grave’s disease, multinodular goitre, thyroid, heart block, AV block, atrioventricular blocks

## Abstract

**Background:**

Atrioventricular block (AVB) is rare in hyperthyroidism (HTH). Little is known about the true prevalence, clinical course, optimal management, and outcomes of different types of AVBs in patients with HTH. To address these uncertainties, we aimed to conduct a systematic review by combining the available literature to provide more meaningful data regarding AVBs in HTH.

**Methods:**

We systematically searched PubMed, Scopus, Embase, and Google Scholar for articles reporting patients who developed AVB in the context of HTH. Data were analysed in STATA 16. The main outcomes included types of AVB, frequency of pacemaker insertion, and resolution of AVB. The systematic review is registered with the International Prospective Register of Systematic Reviews (PROSPERO) with the identification number CRD42022335598.

**Results:**

A total of 56 studies (39 case reports, 12 case series, 3 conference abstracts, 1 retrospective study, and 1 prospective observational study) with 87 patients were included in the analysis, with a mean age of 39.1 ± 17.6 years. Females constituted 65.7% (*n* = 48) of the cohort. Complete heart block (CHB) was the most commonly reported AVB (*N* = 45, 51.7%), followed by first-degree AVB (16.1%) and second-degree AVB (14.9%). Overall, 21 patients underwent pacing. A permanent pacemaker was inserted in one patient with second-degree AVB and six patients with CHB. Mortality was reported in one patient with CHB. The clinical course and management of HTH and AVBs did not differ in patients with CHB or lower-degree blocks. Apart from lower rates of goitre and more use of carbimazole in those who underwent pacing, no differences were found when compared to the patients managed without pacing.

**Conclusion:**

Current data suggest that CHB is the most common type of AVB in patients with HTH. Most patients can be managed with anti-thyroid management alone. Additionally, whether pacemaker insertion alters the clinical outcomes needs further exploration.

## Graphical Abstract

**Figure:**
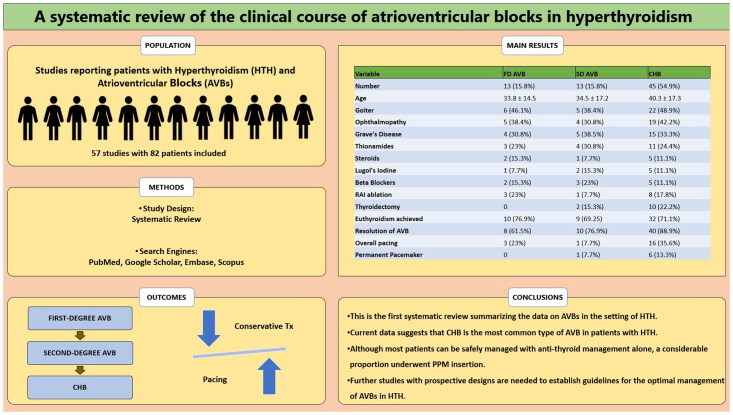

## Introduction

Hyperthyroidism (HTH) is a common clinical condition with a reported prevalence of up to 1.3% [1]. Among other organ systems, HTH can adversely impact cardiac status in multiple ways, including arrhythmias, heart failure, pulmonary hypertension, cardiomyopathy, and valvular dysfunction [2–4]. The types of tachyarrhythmias in HTH vary widely and include sinus tachycardia, premature atrial and ventricular contractions, atrial fibrillation (A.fib), ventricular tachycardia, and ventricular fibrillation. Among these, sinus tachycardia and A.fib are the most common arrhythmias associated with HTH [5]. Atrioventricular blocks (AVBs) of varying degrees have also been reported in patients with HTH.

Little is known about the clinical course of AVBs in association with thyroid disorders. Association between AVB and HTH is rare and has been historically reported, usually with other underlying conditions such as hypercalcemia, viral infection, or co-administration of cardiac medication [6,7]. However, multiple reports describe an association of AVBs in patients with HTH in the absence of any other underlying cause [8,9]. Patients are also reported to present with AVBs as the initial manifestation of HTH [10–12]. Moreover, AVB has been reported to complicate thyroid storm, a life-threatening emergency among patients with HTH [13–17]. Besides overt HTH, where a patient might be considered sick enough to have other organs involved, AVBs are also reported in subclinical HTH [11].

Literature on clinical outcomes of patients with AVB and HTH shows variable results. In cases when AVB is considered to be primarily driven by the HTH state rather than an underlying condition, a restoration of the euthyroid state has shown improvement in the associated conduction defect [8,9,18,19]. Ozcan et al. reported AVB in 21 patients with HTH (8 overt hyperthyroid and 13 subclinical), of which only three patients restored sinus rhythm with the management of HTH alone [20]. To address uncertainties pertaining AVBs in HTH, we aimed to conduct a systematic review by combining the available literature to provide more meaningful and robust evidence regarding AVBs in HTH.

## Materials and methods

### Literature search

A systematic literature search was performed on 14 June 2022, for articles published in English using PubMed/Medline, Google Scholar, Embase, and Scopus for any date up to 14 June 2022. Keywords and mesh terms used to create search syntax included the following: ("Heart block" OR "Atrioventricular block" OR "AV block" OR "AVB") AND ("Thyroid gland" OR "thyroid" OR "thyroids" OR "thyroidal" OR "Hyperthyroidism" OR "thyroiditis” OR "Grave’s disease" OR "Hyperthyroid" OR "Multinodular").

### Study selection

The articles retrieved from the above search term were uploaded to Rayyan.ai for screening. FA and HAK screened the retrieved articles independently. AAK reviewed the disputed articles independently to resolve conflicts. The extracted studies were initially screened from the title and abstract, followed by a full-text screening of the included studies.

### Inclusion criteria

Studies in the English language, reporting primary patient data (case reports, series, observational retrospective, prospective studies, and clinical trials) regarding AVBs (first-degree, second-degree or complete heart block [CHB]) in the context of HTH, were included in the review.

### Exclusion criteria

Exclusion criteria included studies on AVBs in the setting of hypothyroidism and studies in a language other than English. Studies reporting AVBs in patients with HTH amid another established aetiology of AVB (such as cardiac sarcoidosis, Lyme disease, myocardial infarction, electrolyte imbalances, digitalis use, and congenital AVBs) were excluded. Studies describing bundle branch or fascicular blocks in patients with HTH were also excluded. Additionally, review articles with secondary patient data were excluded.

### Quality assessment

**Figure 1.**
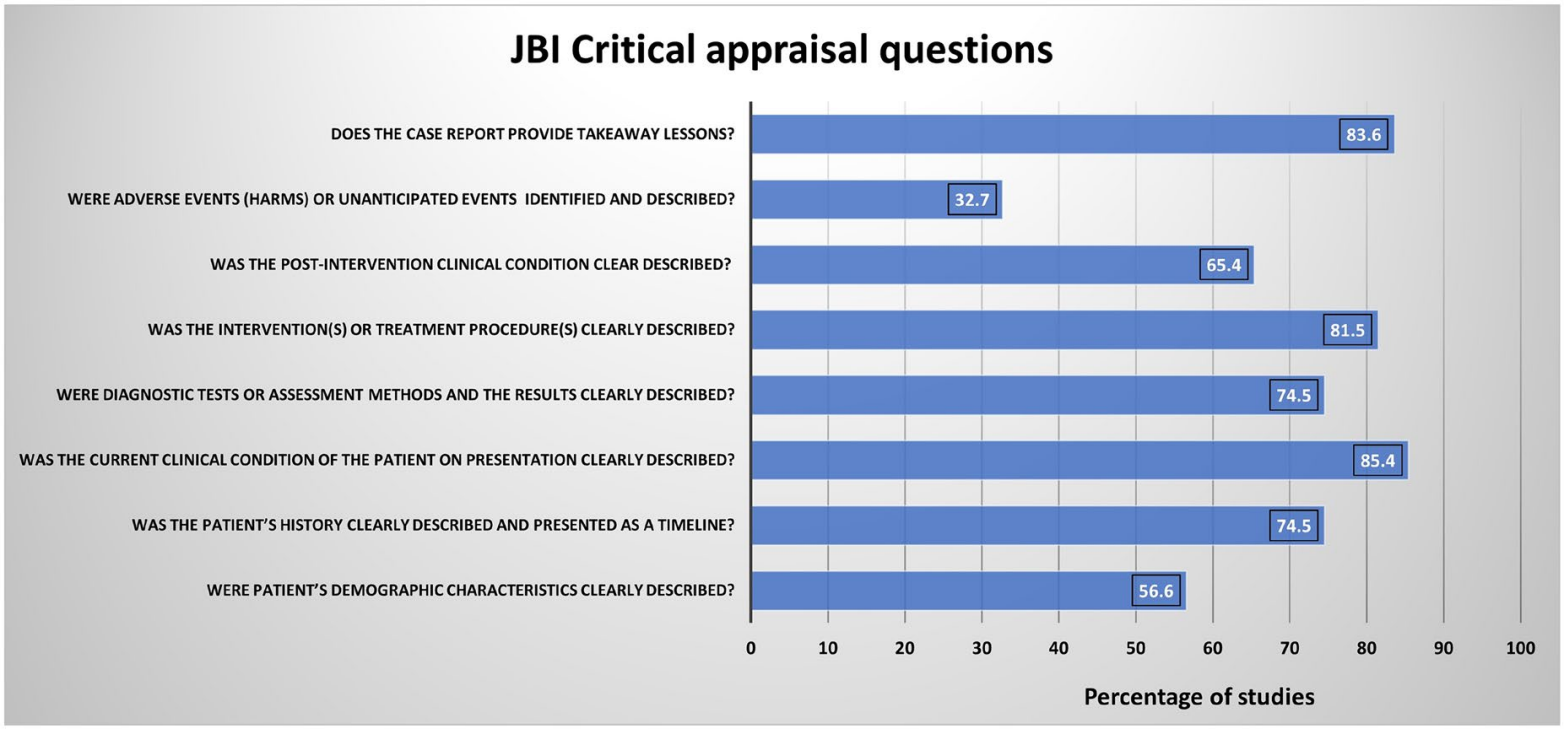
Summary of the quality assessment results of 54 studies (reports and series). The *X*-axis represents the percentage of the studies evaluated, whereas the *Y*-axis represents the questions in the JBI assessment tool against which the studies were assessed.

**Figure 2.**
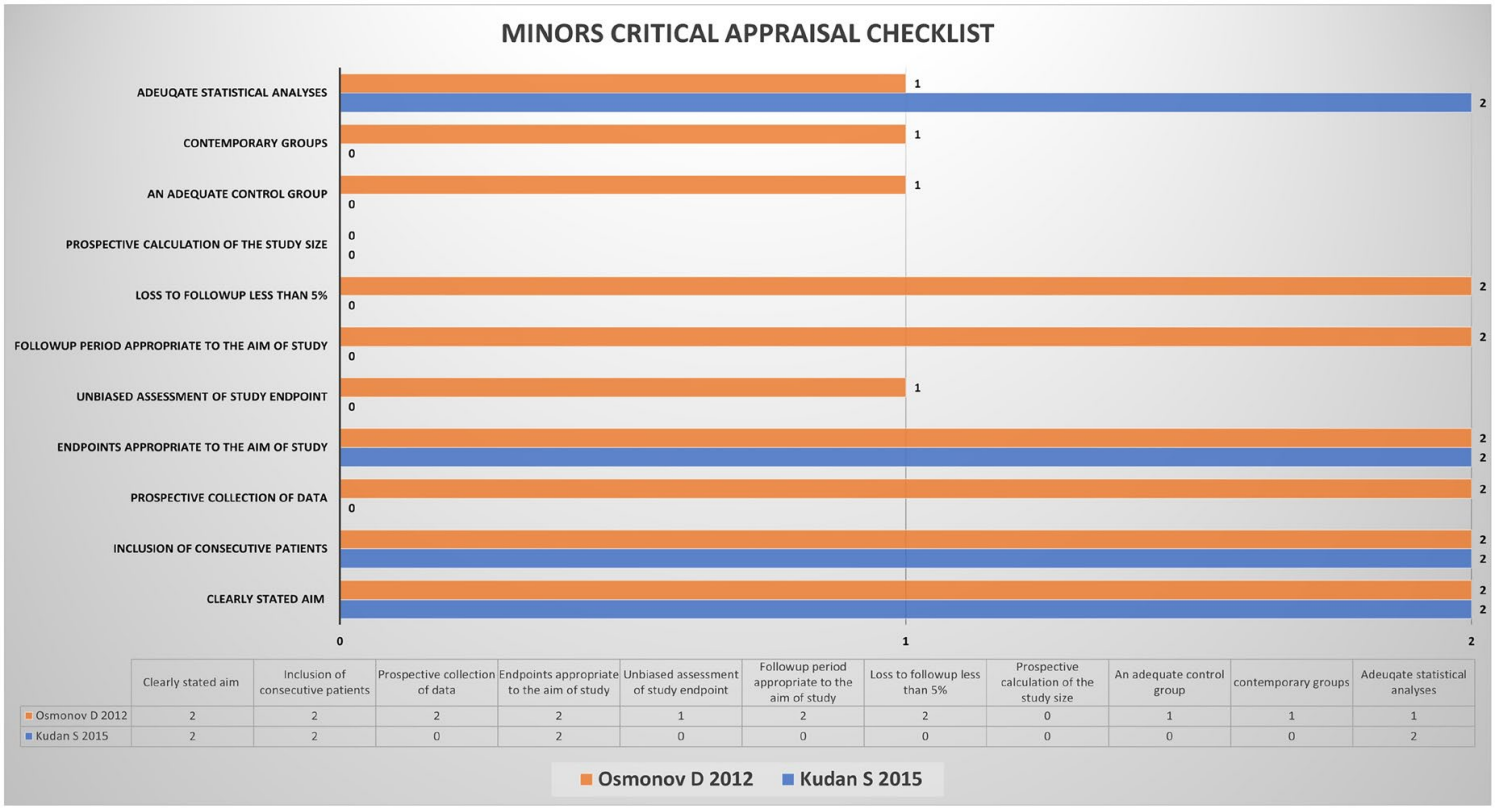
Quality assessment of retrospective and prospective observational studies *via* the methodological index for non-randomized studies (MINORS) scoring system. Orange bars represent data from the prospective study by Osmonov et al. [20], whereas the blue bars represent the data from the retrospective study by Kudan et al. [22]. The *X*-axis denotes the score achieved by the studies, and the *Y*-axis denotes the points against which the studies were scored.

AAK and HAK assessed the quality of the added studies. Case reports and series were assessed using the Joanna Briggs Institute case report appraisal checklist for inclusion in systematic reviews (Figure 1) [21]. Observational studies were assessed using the methodological index for non-randomized studies (MINORS) scoring system (Figure 2) [23]. FA resolved the conflicts *via* an independent review in case of disagreements in the quality assessment.

**Figure 3.**
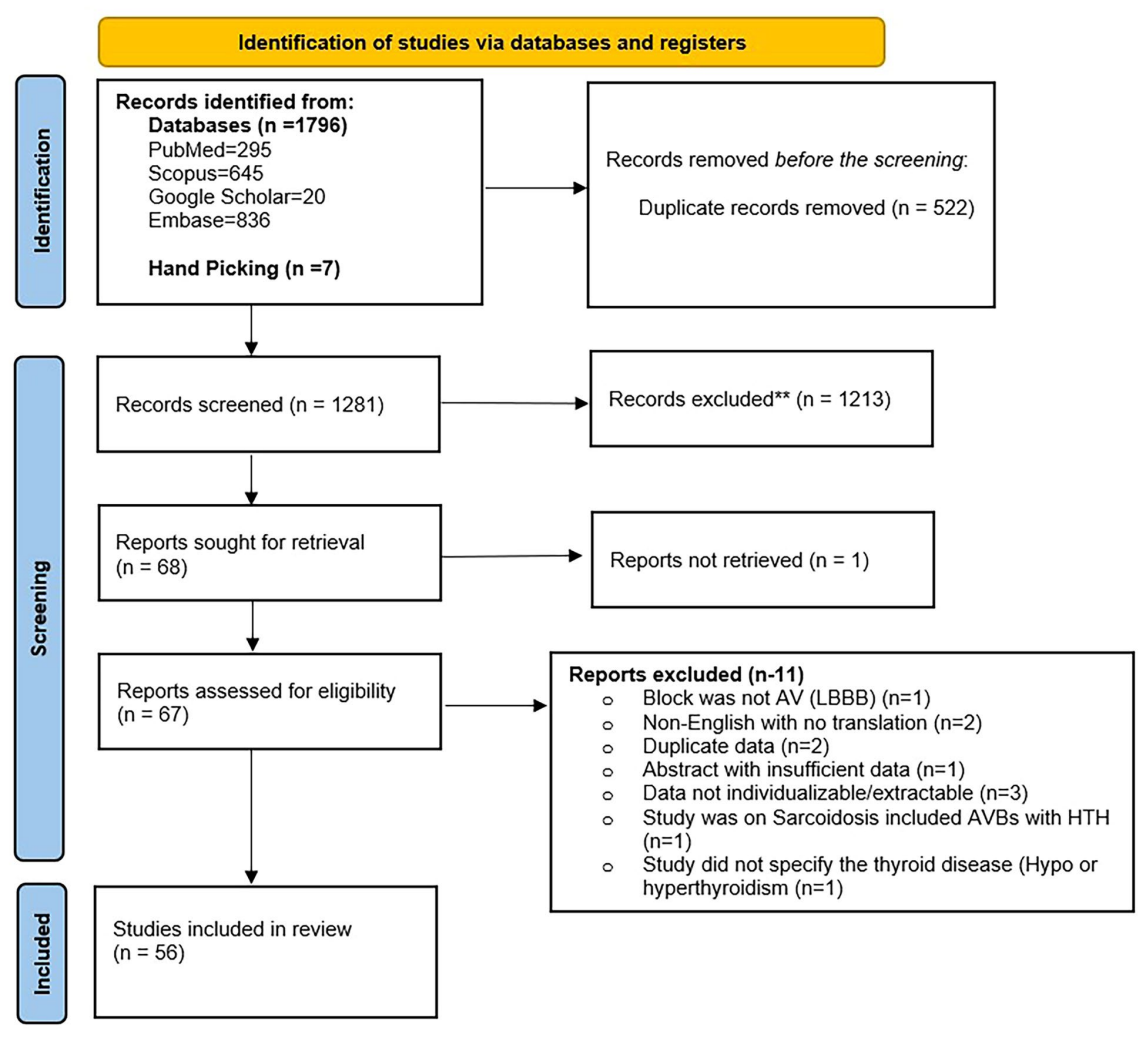
PRISMA flow chart of the screening process of eligible studies with details of included and excluded articles.

### Data collection and statistical analysis

FA, ST, and HAK collected the data independently from the included studies. Data were collected wherever individualizable and reported accordingly in the results. In case of unavailability of individualizable data, the corresponding authors of the studies were contacted *via* email to retrieve the individual patient data. Variables collected included demographic and clinical details, radiological details, management and outcome details of the patients. Data were aggregated into mean with standard deviation or median with interquartile range depending on normality, which was assessed by the skewness of the data, or into numbers with percentages. This systematic review was conducted in keeping with the Preferred Reporting Items for Systematic Reviews and Meta-Analyses (PRISMA) guidelines for reporting systematic reviews [24]. We used independent *t*-test or Mann–Whitney *U* test to analyse continuous variables and chi-square of Fisher’s exact test to analyse categorical variables, as appropriate. All analyses were performed using STATA 16.

## Results

A total of 56 studies (39 case reports, 12 case series, three conference abstracts, one retrospective study, and one prospective observational study) with 87 patients were included in the analysis (Figure 3) (Supplementary file 1).

### Baseline characteristics

The mean age of the patients was 39.1 ± 17.6 years. Females comprised 55.1% (*N* = 48) of the patients, whereas males were 28.7% (25). Gender was not reported in 14 patients (16%). Among the reported comorbidities, hypertension (HTN) (*N* = 5, 5.7%) and heart failure (HF) (*N* = 4, 4.5%) were the most common, followed by A.fib and diabetes mellitus (DM) in 2 patients each (2.3%) (Table 1).

**Table 1.** Baseline characteristics of patients with AVB in HTH.

Variable	Results (*N* = 87)
Age (mean years with SD)	39.1 ± 17.6
Gender	
Male	25 (28.7%)
Female	48 (55.1%)
Not specified	14 (16%)
Comorbidities	
HTN	5 (5.7%)
HF	4 (4.5%)
A.fib	2 (2.3%)
DM	2 (2.3%)

Data are presented as mean (±SD), median (IQR), and numbers (%) as appropriate. AVB: atrioventricular block; HTN: hypertension; DM: diabetes mellitus; HF: heart failure; A.fib: atrial fibrillation; SD: standard deviation; IQR: interquartile range.

### Clinical course and management of HTH

Clinical details of HTH in patients added in the systematic review are presented in Table 2. Among the signs and symptoms of HTH, the most common were goitre (*N* = 35, 47.2%), palpitations (*N* = 29, 39.1%), ophthalmopathy (*N* = 28, 37.8%), tremors and weight loss (*N* = 23, 31% each). Median thyroid-stimulating hormone (TSH) was 0.004 (0.0001–0.018) mIU/L. Thyroid ultrasound was reported in 14 patients, out of which diffuse disease and multinodular goitre (MNG) were seen in 6 (42.8%) patients each, and a single nodule was seen in 2 (14.2%) patients. A radioactive iodine (RAI) uptake scan was reported in 17 patients. Out of these 17, increased uptake was mentioned in 16 (94%) scans. Grave’s disease (GD) was the most common aetiology of HTH, reported in 25 (33.7%) patients, followed by toxic MNG in 3 patients, toxic adenoma and drug-induced thyroiditis in 2 patients each. Treatment of HTH was reported in 74 patients, which included thionamides in 69 (93.2%) patients, RAI ablation in 12 (16.2%) patients, and thyroidectomy in 13 (17.5%) patients. Euthyroidism was achieved in 54 (72.9%) patients after treatment of HTH.

**Table 2.** Clinical details, management, and outcomes of HTH.

Variable	Results
Signs and symptoms (*N* = 74)	
Goitre	35 (47.2%)
Palpitations	29 (39.1%)
Ophthalmopathy	28 (37.8%)
Tremors	23 (31%)
Weight loss	23 (31%)
Sweating	14 (18.9%)
Diarrhea	13 (17.5%)
Heat intolerance	12 (16.2%)
SOB	11 (14.8%)
Syncope	11 (14.8%)
Fever	7 (9.4%)
Dizziness	2 (2.7%)
Fatigue	2 (2.7%)
Laboratory investigations	
TSH (mIU/L), median with IQR	0.004 (0.0001–0.018)
Free T4 (ng/dL), median with IQR (normal range: 0.8–1.8 ng/dL)	4.45 (3.4–6.4)
TRAB + (*N* = 74)	6 (8.1%)
TPO + (*N* = 74)	5 (6.7%)
Thyroid US (*N* = 14)	
Diffuse disease	6 (42.8%)
MNG	6 (42.8%)
Single nodule	2 (14.2%)
RAIU scan (*N* = 17)	
Increased uptake	16 (94%)
Decreased uptake	1 (6%)
Aetiology of HTH (*N* = 74)	
Non-specific	40 (54%)
Grave’s disease	25 (33.7%)
Toxic MNG	3 (4%)
Thyroiditis	2 (2.7%)
Toxic adenoma	2 (2.7%)
Drug-induced thyroiditis	2 (2.7%)
Treatment of HTH (*N* = 74)	
Thionamides (*N* = 69)	
Carbimazole	29 (42%)
PTU	19 (27.5%)
Unspecified	21 (30.4%)
Other Tx	
Thyroidectomy	13 (17.5%)
RAI ablation	12 (16.2%)
Beta-blockers	11 (14.8%)
Lugol’s iodine	9 (12.1%)
Steroids	8 (10.8%)
Outcomes of HTH (*N* = 74)	
Euthyroid post-Tx	54 (72.9%)
Recurrence of HTH	1 (1.3%)
Death	1 (1.3%)

Data are presented as mean (±SD), median (IQR), and numbers (%) as appropriate. HTH: hyperthyroidism; SOB: shortness of breath; TSH: thyroid-stimulating hormone; TRAB: TSH receptor antibody; TPO: thyroid peroxidase; US: ultrasound; MNG: multinodular goitre; RAIU: radioactive iodine uptake; PTU: propylthiouracil; Tx: treatment; RAI: radioactive iodine; SD: standard deviation; IQR: interquartile range.

### Clinical course and management of AVBs

CHB was the most common type of AVB reported in the patients (*N* = 45, 51.7%), followed by first-degree AVB in 14 (16.1%) and second-degree AVB in 13 (14.9%). The level of AVB was not specified in 15 (17.2%) patients. Pacing was required in 21 (28.4%) patients. Of these 21 patients, two underwent transcutaneous pacing, nine underwent transvenous pacing, and seven underwent permanent pacemaker (PPM) insertion, whereas the pacing method was not specified in three patients. Atropine use was reported in 13 (17.5%) patients. The commonest reported complications were a vasopressor requirement in seven patients and intubation in five. Only one death was reported: a 53-year-old female with GD complicated with a CHB. The patient had nausea and vomiting with a reduction in pulse rate from 116 to 70 beats per minute. Post-mortem findings showed cellular infiltration of the AV node. Further analysis showed the presence of gram-positive bacteria in the ventricular portion of the AV node [6]. The outcomes of AVB were found in 73 patients. A resolution of the AVB was reported in 60 (82.2%) patients, whereas six patients (8.2%) had persistent AVB. A progression of the initial AVB to any higher degree of AVB was reported in 3 (4.1%) patients, and progression to CHB was reported in 3 (4.1%) patients, whereas one patient had a recurrence of AVB after resolution (Table 3).

**Table 3.** Clinical details of AVBs in patients with HTH.

Variable	Results
Type of AVB (*N* = 87)	
Complete heart block	45 (51.7%)
First-degree AVB	14 (16.1%)
Mobitz Type I AVB	6 (6.9%)
Mobitz Type II AVB	1 (1.1%)
Second-degree AVB unspecified	6 (6.9%)
Unspecified level of AVB	15 (17.2%)
Pacing (*N* = 21)	
Transcutaneous pacing	2 (9.5%)
Transvenous pacing	9 (42.9%)
Permanent pacemaker	7 (33.3%)
Unspecified type of pacing	3 (14.3%)
Atropine	13 (17.5%)
Outcomes of AVB (*N* = 74)	
Resolution of AVB	60 (82.2%)
Persistent AVB	6 (8.2%)
Progression to higher-degree AVB	3 (4.1%)
Became CHB	3 (4.1%)
Recurrence of AVB	1 (1.3%)
Complications (*N* = 74)	
Vasopressors	7 (9.4%)
Intubation	5 (6.7%)
Impaired LVF	1 (1.3%)
Ventricular fibrillation	1 (1.3%)
Cardiogenic shock	1 (1.3%)
Mortality (*N* = 87)	1 (1.1%)

Data are presented as mean (±SD), median (IQR), and numbers (%) as appropriate. AVB: atrioventricular block; HTH: hyperthyroidism; CHB: complete heart block; LVF: left ventricular function.

### Follow-up data of the patients

Out of 56 studies, follow-up (FU) details were available in 38 patients. The median duration to FU was 150 days (IQR 60–210). FUs were done as early as 7 days after discharge to as long as 4 years after discharge [6,25]. Twenty (52.6%) of the 38 patients with FU remained euthyroid, one (2.6%) had a persistent hyperthyroid state, one died during the initial hospital stay, and the thyroid status was not reported in rest. Concerning the FU details of the AVBs, 24 patients (63.1%) were reported to have sinus rhythm. In one patient, the initial second-degree AVB converted to first-degree AVB upon FU, and one patient had persistence of bradycardia–tachycardia syndrome [11,26].

### Outcome analysis

#### Clinical details of the patients based on the type, management, and outcomes of AVB

Table 4 summarizes the differences in the clinical characteristics, management and outcomes among the patients with HTH based on the type of AVB (CHB compared to other lower-degree AVBs). None of the differences reached statistical significance.

**Table 4.** Characteristics and outcomes of patients based on complete vs. other AVBs (*N* = 72).

Variable	Complete AVB *N* = 45	Other AVBs *N* = 27	Significance
Age (mean with SD)	40.3 ± 17.3	34.2 ± 15.6	0.14
Gender			
Female	30 (66.7%)	16 (59.2%)	0.8
Male	15 (33.3%)	9 (33.3%)	
Ophthalmopathy	19 (42.2%)	9 (33.3%)	0.5
Goitre	22 (48.9%)	11 (40.7%)	0.7
Grave’s disease	15 (33.3%)	9 (33.3%)	0.8
Laboratory workup (median with IQR)			
TSH	0.004 (0.0001–0.08)	0.006 (0.00002–0.02)	1
Free T4 (normal range: 0.8–1.8 ng/dL)	4.7 (3.7–6.4)	4 (2.05–5.5)	0.4
Treatment of HTH			
Carbimazole	21 (46.6%)	7 (25.9%)	0.06
Propylthiouracil	11 (24.4%)	8 (29.6%)	0.6
RAI ablation	8 (17.8%)	4 (14.8%)	0.7
Surgery	10 (22.2%)	2 (7.4%)	0.09
Euthyroid post-treatment	32 (71.1%)	20 (74.1%)	0.8
Pacing (any type)	16 (35.5%)	4 (14.8%)	0.07
Permanent pacemaker insertion	6 (13.3%)	1 (3.7%)	0.2
Persistent AVB	2 (4.4%)	4 (14.8%)	0.1
Resolution of AVB	40 (88.9%)	18 (66.7%)	0.07
Intubation	4 (8.9%)	1 (3.7%)	0.4
Vasopressor requirement	5 (11.1%)	2 (7.4%)	0.6
Mortality	1 (2.2%)	0	0.4

Results are reported based on the availability of outcome data. Data are presented as mean (±SD), median (IQR), and numbers (%) as appropriate. AVB: atrioventricular block; TSH: thyroid-stimulating hormone; HTH: hyperthyroidism; RAI: radioactive iodine; SD: standard deviation; IQR: interquartile range.

Table 5 summarizes the clinical details of the patients categorized into those who underwent pacing vs. those who did not undergo pacing (conservative management). The mean age was 38.3 ± 2.5 years in the no-pacing group compared to 40.8 ± 3.9 years in the pacing group (*p* = 0.6). No-pacing group comprised 64.2% (34) females and 33.9% (18) males. The pacing group had 66.7% (14) females and 33.3% (7) males (*p* = 0.9). Ophthalmopathy was reported in 21 (39.6%) patients in the no-pacing group and 7 (33.3%) patients in the pacing group (*p* = 0.6). A goitre was present in 15 (28.3%) patients in the no-pacing group, compared to 6 (28.5%) patients in the pacing group (*p* = 0.03). GD was more commonly reported aetiology in the pacing group (47.6% vs. 28.3%) (*p* = 0.1). Carbimazole use was significantly more in patients who underwent pacing (66.7%) compared to those who did not do pacing (28.3%) (*p* = 0.006). Other treatments of HTH did not significantly differ in the two groups. CHB was present in 54.7% of patients in the no-pacing group, compared to 76.2% in the pacing group (*p* = 0.07). The persistence, progression and resolution of AVBs did not significantly differ in the two groups.

**Table 5.** Characteristics and outcomes of patients based on pacing or no-pacing (*N* = 74).

Variable	No-pacing	Pacing	Significance
	*N* = 53	*N* = 21	
Age (mean with SD)	38.3 ± 2.5	40.8 ± 3.9	0.6
Gender			
Female	34 (64.2%)	14 (66.7%)	0.9
Male	18 (33.9%)	7 (33.3%)	
Ophthalmopathy	21 (39.6%)	7 (33.3%)	0.6
Grave’s disease	15 (28.3%)	10 (47.6%)	0.1
Laboratory workup			
TSH (median with IQR)	0.003 (0.006–0.01)	0.008 (0.00003–0.03)	0.6
Free T4 (normal range: 0.8–1.8 ng/dL)	4.8 (3.2–6.7)	4.3 (3.7–6.4)	0.9
Treatment of HTH			
Carbimazole	15 (28.3%)	14 (66.7%)	**0.006**
Propylthiouracil	13 (24.5%)	6 (28.6%)	0.9
Steroids	5 (9.4%)	3 (14.3%)	0.2
RAI ablation	10 (18.9%)	2 (9.5%)	0.2
Surgery	11 (20.8%)	2 (9.5%)	0.2
Euthyroid post-treatment	34 (64.2%)	19 (90.5%)	0.1
Type of AVB			
Complete heart block	29 (54.7%)	16 (76.2%)	0.07
Other AVBs	22 (41.5%)	4 (19%)	
Persistent AVB	3 (5.7%)	3 (14.3%)	0.2
Progression to higher degree AVB	3 (5.7%)	0	0.25
Resolution of AVB	42 (79.2%)	18 (85.7%)	0.6
Intubation	2 (3.8%)	3 (14.3%)	0.13
Vasopressor requirement	3 (5.7%)	4 (19%)	0.1
Mortality	1 (1.9%)	0	0.5

Data are presented as mean (±SD), median (IQR), and numbers (%) as appropriate. TSH: thyroid-stimulating hormone; HTH: hyperthyroidism; RAI: radioactive iodine; AVB: atrioventricular block; SD: standard deviation; IQR: interquartile range. Bold values represent statistically significant *P*-value

Table 6 summarizes the differences among clinical characteristics and management strategies of HTH and AVBs based on the resolution of AVB. Carbimazole use was significantly higher in those patients who had a resolution of AVB (45%) compared to those in whom AVB did not resolve (15.4%) (*p* = 0.03). Euthyroidism post-HTH treatment was seen more in patients with AVB resolution than with unresolved AVBs (76.7% vs. 53.8%) (*p* = 0.051). None of the other factors were significantly different in the two groups.

**Table 6.** Characteristics and outcomes of patients based on the resolution of AVB (*N* = 73).

Variable	No resolution	Resolution	Significance
	*N* = 13	*N* = 60	
Age (mean with SD)	38.5 ± 4.9	39.4 ± 2.4	0.9
Gender			
Female	9 (69.2%)	39 (65%)	0.8
Male	4 (30.7%)	20 (33.3%)	
Ophthalmopathy	5 (38.4%)	23 (38.3%)	0.9
Grave’s disease	6 (46.1%)	19 (31.6%)	0.3
Laboratory workup			
TSH (median with IQR)	0.003 (0.006–0.01)	0.008 (0.00003–0.03)	0.6
Free T4 (normal range: 0.8–1.8 ng/dL)	4.8 (3.2–6.7)	4.3 (3.7–6.4)	0.9
Treatment of HTH			
Carbimazole	2 (15.4%)	27 (45%)	**0.03**
Propylthiouracil	3 (20%)	16 (26.6%)	0.7
Steroids	2 (15.4%)	6 (10%)	0.6
RAI ablation	2 (15.4%)	10 (16.6%)	0.8
Surgery	2 (15.4%)	11 (18.3%)	0.7
Euthyroid post-treatment	7 (53.8%)	46 (76.7%)	0.051
Type of AVB			
Complete heart block	5 (38.4%)	40 (66.7%)	0.07
Other AVBs	8 (61.5%)	20 (33.3%)	
Intubation	0	5 (8.3%)	0.3
Vasopressor requirement	1 (7.7%)	6 (10%)	0.7

Results are reported based on the availability of outcome data. Data are presented as mean (±SD), median (IQR), and numbers (%) as appropriate. AVB: atrioventricular block; TSH: thyroid-stimulating hormone; HTH: hyperthyroidism; RAI: radioactive iodine; SD: standard deviation; IQR: interquartile range. Bold values represent statistically significant *P*-value

## Discussion

To the best of our knowledge, this study represents the first systematic review investigating the clinical course and outcomes of AVB in the context of HTH. CHB was the most common type of AVB reported in patients with HTH (reported in 51.7%). Patients with CHB were older than those with other AVBs. Of the 45 patients with CHB, 16 (35.5%) required pacing, of which 6 (13.3%) patients required a PPM. Interestingly, the resolution of AVB was highest in patients with CHB in the setting of HTH (88.9%). We found no differences in the clinical characteristics and clinical course of the patients based on the type of AVB, pacing vs. no-pacing, and resolution of AVB compared to no resolution.

The pathophysiology of AVBs in the setting of HTH remains unknown, mainly due to the small proportion of patients who develop AVBs in a hyperthyroid state, making it impractical to study the condition in larger prospective designs. Some leading theories proposing the mechanism of AVBs in HTH include inflammation of the myocardium adjacent to AV nodes, and exhaustion of the autonomic nervous system due to sustained tachycardia in earlier phases [19,27,28]. TSH receptor mRNA is expressed in different tissues, including the heart, gonads, skeletal muscles, and fat cells [29]. These receptors may play a central role as a protagonist of myocardial inflammation, similar to thyroid ophthalmopathy in GD, eventually causing a nodal block. Thyrotoxic periodic paralysis (TPP), characterized by sudden paralysis and hypokalaemia, may cause AVB [30]. The AVB is thought to be a consequence of potassium channel dysregulation in TPP. None of the publications included in this review exhibited TPP. Hence, it is unlikely to be the pathogenetic mechanism behind AVBs in HTH. The literature lacks clarity due to a deficiency in extensive studies validating the proposed mechanisms.

HTH can cause dysfunction of the myocardium due to severe and toxic effects of the thyroid hormone, which can eventually exhaust the cardiac tissue, resulting in HF [4]. A similar theory can be proposed for its exhaustive effects on the cardiac conduction system, leading to eventual dysfunction. However, the literature lacks enough data to support the theory. AVBs are also reported in subclinical HTH, further weakening the hypothesis of the toxic effects of uncontrolled thyroid hormone [11]. Sun et al. investigated the effects of thyroid hormone on the cardiac conduction system in rats, revealing the genomic and non-genomic effects of thyroid hormone in controlling the duration of action potential and repolarization in the sinoatrial nodes [31]. A higher-than-normal presence of thyroid hormone in the myocytes regulating the pacemaker activity of the heart can alter the transcription of the pacemaker genes, leading to tachyarrhythmias at one end and AVBs on the other [32].

Beta-blockers (BBs) have been used for decades in the management of HTH, for their efficacy in alleviating catecholamine-mediated symptoms such as palpitations, tremors, and tachycardia [33]. BBs are known to cause bradyarrhythmia owing to their chronotropic effects. This is more commonly seen with cardio-selective BB. The majority of information available on the bradyarrhythmia that can occur with BB use comes from studies on HF. However, these studies often have limitations, as bradycardia is typically considered an exclusion criterion in such studies. In this review, many patients were reported to have used BB to treat AVBs [7,18,34–40]. However, some authors reported the presence of AVBs before the diagnosis of HTH was made and hence before the BBs were initiated [38,39]. There was only one case where the authors mentioned the occurrence of AVB after initiating treatment for HTH, and raised the possibility of AVB being secondary to BBs rather than HTH itself [18]. Many studies associated the AVBs with HTH and not with BB use. Nevertheless, the ambiguities concerning BB use in patients with AVB in the setting of persist and only further prospective research can clarify the uncertainty.

Recent reviews on acquired AVBs in the young population have argued that patients might be overtreated with interventions including PPM insertion, whereas the outcomes might not be different if treated conservatively, managing the underlying condition [41]. Ozcan et al. published the only prospective study on 21 patients with HTH and heart blocks, of which 12 had AVBs [20]. The authors divided the patients into those who improved with HTH management (hence defining it as HTH-induced AVBs) and those who did not improve with HTH management (defining them as AVBs unrelated to HTH). 20 of the 21 patients in the study underwent PPM insertion. The authors concluded that all patients with AVBs in the setting of HTH require PPM. Our review shows different results, with a very small number of patients requiring PPM, which did not alter the outcomes. Ozcan et al. screened patients with AVBs to identify those with HTH. As cohorts of patients with AVB are usually older and sicker (as most AVBs occur in elderly with multiple comorbid conditions and usually with ischaemic heart disease), the resultant cohort of AVB in thyroid disorders had a mean age of 68.2 years. This might have created a bias in the results and conclusions related to using PPM in AVBs with HTH. On the other hand, patients with HTH are usually younger when diagnosed, albeit more symptomatic than older populations [42]. Choosing the cohort can result in different age groups, which might influence the results. Selecting patients with HTH and identifying AVBs among them can probably give more reliable results as this population is young and relatively free from other comorbid conditions (except HTH).

We found that treatment with carbimazole was associated with higher rates of resolution of AVBs in patients with HTH (*p* = 0.03). This finding could be multifactorial. Firstly, patients who received carbimazole had more CHBs than those who did not. Additionally, patients on carbimazole underwent pacing more than those who did not receive carbimazole. This could have influenced the positive outcome in patients taking carbimazole. However, some data exist concerning the effects of carbimazole or methimazole on cardiac conduction. Research has demonstrated improvements in cardiac conduction in patients with HTH treated with carbimazole or methimazole [43]. Additionally, there is indirect evidence suggesting that abrupt cessation of carbimazole may lead to destabilization of the cardiac membrane [44].

The principal strength of this study is the accumulation of data on AVBs in the setting of HTH for the first time in the form of a comprehensive systematic review. Due to the small proportion of patients that develop AVBs in a hyperthyroid state, it seems impractical to study the condition in larger prospective designs. Hence summarizing the previously published data will add to understand the phenomenon with clarity. However, the limitations of this study which are inherent to systematic reviews of observational data, need to be acknowledged. This review included only those patients with HTH who developed AVBs; hence prevalence or burden of AVBs in the setting of HTH could not be calculated. Secondly, publication bias has to be considered, as unpublished work might have influenced the review results. Thirdly, various factors that the treating physicians would have considered to decide on PPM insertion in AVBs might not have been reported and hence not summarized in this review. Fourthly, given the retrospective nature of the included studies, a causality between HTH and AVBs cannot be confirmed. Another important limitation is the lack of genetic data, which could have provided deeper insights into the genetic predispositions that might influence the incidence or severity of AV blocks in hyperthyroid patients. Nevertheless, this review establishes the existence of AVBs in the context of HTH with a seemingly higher burden of CHBs compared to other degrees of AVB. This review opens doors to future research on AVBs in HTH, focusing on factors that can predict a need for PPM insertion.

## Conclusion

Current data suggest that CHB is the most common type of AVB in patients with HTH. However, CHB tends to follow a benign course and does not have significantly different outcomes compared to lower-degree AVBs in HTH. Most patients can be managed with anti-thyroid management alone, and only a small proportion may need a PPM insertion. Additionally, whether pacemaker insertion alters the clinical outcomes needs further exploration. Further studies are needed to establish guidelines for the optimal management of AVBs in HTH.

## Supplementary Material

Supplemental Material

## Data Availability

Data sharing is not applicable.

## Review registration link

https://www.crd.york.ac.uk/PROSPERO/display_record.php?RecordID=335598
