# Supplementary material for: A systematic review of the clinical characteristics and course of atrioventricular blocks in hyperthyroidism
Source: Ann Med. 2024 Jun 21;56(1):2365405. doi: 10.1080/07853890.2024.2365405 (PMC11195459; doi:10.1080/07853890.2024.2365405)
Supplement: Supplemental Material [file IANN_A_2365405_SM6329.pdf]

Studies added in the systematic review (1-56)

1. Adesokan A, Vigneswaran T, Ajzensztejn M, Mathur S. Atrioventricular block: an unusual complication of Graves' disease. BMJ Case Rep.2017.
2. Aguilar AI, oval MS. Hyperthyroidism-induced heart block: A case series in the Philippine general hospital. Thyroid.1:A76.
3. Al Bannay R, Husain A, Khalaf S. Complete heart block in thyrotoxicosis, is it a manifestation of thyroid storm? A case report and review of the literature. Case Rep Endocrinol. 2012;2012:318398.
4. Ariturk Z, Tekbas E, Soydinc S, Yazici M, Islamoglu Y, Cil H, et al. An unusual presentation of hyperthiroidism: Atrioventricular complete heart block. Heart Surgery Forum.1:S106-S7.
5. Ashawesh K, Kulambil Padinjakara RN, Murthy NPN, Sankar S, Syed A. Severe dilated cardiomyopathy and hyperthyroidism. Endocrine Abstracts. 2009;19:P378.
6. Atri SK, Chugh SN, Goyal S, Chugh K. Reversible atrioventricular blocks in thyroid storm. Journal of Association of Physicians of India. 2011;59(3):178-9.
7. Bannay RA, Husain A, Khalaf S. Complete Heart Block in Thyrotoxicosis, Is It a Manifestation of Thyroid Storm? A Case Report and Review of the Literature. Case Reports in Endocrinology. 2012.
8. Boccal, ro C, Lopez L, Boccal, ro F, Lavis V. Electrocardiographic changes in thyrotoxic periodic paralysis. American Journal of Cardiology. 2003;91(6):775-7.
9. Bourne G, Hartley P. Heart-Block Influenced by Inspiration in a Case of Graves' Disease. Journal of the Royal Society of Medicine. 1928;21(3):322.
10. Boysan SN, Olgar S, Aksu E, Cabioglu DE, Sahin E, Temizdemir H, et al. Kearns-sayre syndrome: A case report presenting endocrine features and associated with bartter-like phenotype. Endocrine Reviews Conference: 97th Annual Meeting and Expo of the Endocrine Society, ENDO. 2015;36.
11. Cameron JDS, Hill IGW. Heart Block in Toxic Goitre: A Report of Two Cases. Edinb Med J. 1932;39(1):37-50.
12. Campus S, Rappelli A, Malavasi A, Satta A. Heart block and hyperthyroidism. Report of two cases. Arch Intern Med.135(8):1091-5.
13. Celebi AS, Amasyali B. Reversible first-degree atrioventricular block due to hyperthyroidism. [Turkish]. Turk Kardiyoloji Dernegi Arsivi.45:275-7.
14. Crăcană I, Vasilcu TF, Mardare A, Alexa ID, Marcu DT. SEVERE AMIODARONE-INDUCED BRADICARDIA CONCEALES SICK SINUS SYNDROME: CASE REPORT. Rev Med Chir Soc Med Nat Iasi.120(1):110-3.
15. Dave JA, Ross IL. Complete heart block in a patient with Graves' disease (Thyroid (2008) 18, (1329-1331)). Thyroid. 2009;19(2):207.
16. Davis AC, Smith HL. Complete heart-block in hyperthyroidism following acute infections: A report of six cases with necropsy findings in one case. American Heart Journal. 1933;9(1):81-9.
17. El-Harasis MA, DeSimone CV, Stan MN, McLeod CJ, Noseworthy PA. Graves' disease-induced complete heart block and asystole. HeartRhythm Case Rep.4(3):105-8.
18. Eom YS, Oh PC. Graves' Disease Presenting with Complete Atrioventricular Block. Case Rep Endocrinol. 2020;2020:6656875.
19. Eraker SA, Wickamasekaran R, Goldman S. Complete heart block with hyperthyroidism. Jama.239(16):1644-6.
20. Grenadier E, Keidar S, Alpan G, Viener A, Palant A. Complete atrioventricular block, shock, and hyperkalemia induced by toxic adenoma of the thyroid gland. Heart Lung.10(1):105-8.

21. Gupta A, Arora S. Atriov entricular heart blocks in thyrotoxicosis. Journal of the American College of Cardiology.1:A738.
22. Ho S, Eng P, Ding Z, Fok A, Khoo D. Thyroid storm presenting as jaundice and complete heart block. ANNALS-ACADEMY OF MEDICINE SINGAPORE. 1998;27:748-51.
23. Jalal S, Khan KA, Rauoof MA, Jan VM, Lone NA, Rather HA, et al. Thyrotoxicosis presenting with complete heart block. Saudi Med J.25(12):2057-8.
24. Karakaş CY, Topaloğlu C, Canbolant E, Seyfeli E, Akgül F. Hyperthyroidism as a rare cause of complete AV block. Anadolu Kardiyol Derg.9(1):67-8.
25. Kausel A, Korniyenko A, Sandhu G. Bradyarrhythmia as a presenting feature of subclinical hyperthyroidism. QJM: An International Journal of Medicine. 2011;105(5):461-2.
26. Kernoff LM, Rossouw JE, Kennelly BM. Complete heart block complicating thyrotoxicosis. S Afr Med J.47(12):513-5.
27. Khan MZ, Qureshi F, Mahfooz F, Brown K. Grave Danger: Rare Case of Complete Heart Block Secondary to Graves Disease. Journal of the American College of Cardiology. 2022;79(9 Supplement):2512.
28. Kramer MR, Shilo S, Hershko C. Atrioventricular and sinoatrial block in thyrotoxic crisis. Br Heart J.54(6):600-2.
29. Krishnamoorthy S, Narain R, Creamer J. Unusual presentation of thyrotoxicosis as complete heart block and renal failure: a case report. Journal of Medical Case Reports. 2009;3(1):1-4.
30. Kudan S, Lal M, Angral R. The prevalence of cardiovascular abnormalities in thyrotoxicosis – A cross sectional study. Journal of Medicine (Bangladesh). 2015;16(2):69-72.
31. Kuo YC, Tseng YT, Lee TI, Hsieh MH. Chronic bifascicular block with intermittent complete atrioventricular block induced by hyperthyroidism. Int J Cardiol. 2006;110(3):407-10.
32. Kurnick JE, Hartman CR, Floyd GD, Spicer MJ, Nelson WP. Wenckebach phenomenon in thyrotoxicosis with hypercalcemia. Rocky Mt Med J.70(10):46-7.
33. Levy DW. Complete heart block complicating thyrotoxicosis. S Afr Med J.47(20):853.
34. Maher CC, Sanders A, Plice SG, Wosika PH. A syndrome of exophthalmic goiter, acute rheumatic carditis, and heart block. American Heart Journal. 1939;17(6):742-8.
35. Meles E. Sinoatrial blocks in hyperthyroidism associated with syncope, treated with beta-blockers. A case of paradoxical treatment. Giornale Italiano di Cardiologia. 2011;1):56S-7S.
36. Miller RH, Corcoran FH, Baker WP. Second and third degree atrioventricular block with Graves' disease: a case report and review of the literature. Pacing Clin Electrophysiol.3(6):702-11.
37. Muenthongchin N, Vejjajiva A, Jumbala B. Disturbance of atrio-ventricular conduction in a patient with thyrotoxic periodic paralysis. J Med Assoc Thai.53(11):814-9.
38. Muggia AL, Stjernholm M, Houle T. Complete heart block with thyrotoxic myocarditis. Report of a case. N Engl J Med.283(20):1099-100.
39. Ortmann C, Pfeiffer H, Du Chesne A, Brinkmann B. Inflammation of the cardiac conduction system in a case of hyperthyroidism. International Journal of Legal Medicine. 1999;112(4):271-4.
40. Osman F, Ayuk J, Dale J, Franklyn JA, Gammage MD. Thyrotoxicosis with heart block. J R Soc Med. 2001;94(7):346-8.
41. Osmonov D, Ozcan KS, Erdinler I, Yildirim E, Altay S, Turkkan C, et al. Atrioventricular block in patients with thyroid dysfunction: Prognosis after treatment with the hormone supplementation or antithyroid medication. [Turkish, English]. Turk Kardiyoloji Dernegi Arsivi.1:204.
42. Ralapanawa U. High Grade AV Block and dynamic ECG changes complicating thyrotoxicosis in autoimmune thyroiditis. 2017.
43. Rosenblum R, Delman AJ. FIRST-DEGREE HEART BLOCK ASSOCIATED WITH THYROTOXICOSIS. Arch Intern Med.112:488-90.

44. Roy S, Goswamy V, Singh H, Portales I, Sheppard T, Feitell S. Nivolumab-induced thyroid storm and cardiogenic shock in a patient with metastatic melanoma. Journal of the American College of Cardiology Conference: 67th Annual Scientific Session of the American College of Cardiology and i2 Summit: Innovation in Intervention, ACC. 2018;71(11).
45. Sampana AG, Jasul Jr GV. High grade AV block complicating hyperthyroidism: A case report. Phillippine Journal of Internal Medicine. 2010;48(2):38-40.
46. Sataline L, Donaghue G. Hypercalcemia, heart-block, and hyperthyroidism. Jama.213(8):1342.
47. Sawano K, Hiroshima S, Shibata N, Nyuzuki H, Ogawa Y, Nagasaki K. A case of complete atrioventricular block after initiation of methimazole in a patient with Graves' disease associated with Down's syndrome previously undergoing cardiac surgery. Hormone Research in Paediatrics. 2021;94:405-6.
48. Širanec M, Magage S, Válek M, Marek J, Šimek J, Bělohlávek J, et al. Third degree atrioventricular block as a rare complication of Graves' thyrotoxicosis. Cor et Vasa. 2019;6(6):629-33.
49. Stern MP, Jacobs RL, Duncan GW. Complete Heart Block Complicating Hyperthyroidism. JAMA. 1970;212(12):2117-9.
50. Sunardi AEP, Gunawan EJ, Benny S, a AL. Total AV block in hyperthyroid. Journal of Arrhythmia.35:25.
51. Szczeklik A, Nowicka J. Atrioventricular block in hyperthyroidism. Pol Med Sci Hist Bull.14(3):110-1.
52. Topaloglu S, Topaloglu OY, Ozdemir O, Soylu M, Demir AD, Korkmaz S. Hyperthyroidism and complete atrioventricular block--a report of 2 cases with electrophysiologic assessment. Angiology.56(2):217-20.
53. Vennard K, Gilbert MP. Thyroid Storm and Complete Heart Block after Treatment with Radioactive Iodine. Case Rep Endocrinol. 2018;2018:8214169.
54. Wang HF, Tsai SC, Pan MS, Shiao CC. Complete heart block during potassium therapy in thyrotoxic periodic paralysis. Journal of Emergency Medicine. 2013;44(1):61-4.
55. Yusoff K, Khalid BA. Conduction abnormalities in thyrotoxicosis--a report of three cases. Ann Acad Med Singap. 1993;22(4):609-12.
56. Zargar AH, Bashir MI, Wani AI, Laway BA, Masoodi SR, Lone NA, et al. Reversible complete heart block in Grave's disease. J Assoc Physicians India.47(11):1120-1.
